# Cases of Fatal but Chronic Termination of Inguinal Hernia, from Simple Obstruction

**Published:** 1841-10-30

**Authors:** W. W. Gerhard


### ORIGINAL COMMUNICATIONS.

*Cases of Fatal but Chronic termination of Inguinal Hernia, from simple obstruction.* By W. W. Gerhard, M. D.

It is rare that hernia produces obstruction to the passage of the fœcal matter through the bowels to a sufficient degree to cause severe and even fatal symptoms, without the mortification of the gut. These cases are, however, from time to time met with, and may cause much embarrassment to the physician; for the nature of the symptoms is such that they are not always supposed to belong to surgical affections. Two fatal cases of this kind have come under my notice.

*Case* 1. The first was in the month of October, 1835. The patient was labouring under an irreducible inguinal hernia, of moderate size, (not exceeding that of a small pullet's egg;) he was affected with nausea and vomiting, with obstinate constipation, beginning gradually and increasing for several weeks, when death took place. The tumour was perfectly free from pain, and very uniform in size and shape: the quantity of fœcal matter passed was small, but of healthy appearance and consistence. Vomiting occurred very frequently and finally consisted merely of the substances taken into the stomach, which were rejected almost as soon as swallowed.

The existence of the hernial tumour, notwithstanding the mildness and slowness of the symptoms, induced me to call a consultation of surgeons, and to desire putting the case into their hands. The gentlemen who were consulted in the case declined resorting to an operation or other surgical means; all other modes of treatmeut proved unavailing.

On examination after death, the portion of the bowel contained in the hernial sac was found to be the ileum; the included fold was full of fœcal matter, but neither sphacelated nor inflamed; above the hernia the bowels were extremely distended by their ordinary contents, and below, they were shrunken and contracted.

There was, therefore, in this case, an obstruction sufficient to cause death without strangulation. It was of course greatly to be regretted that no operation was thought proper by the gentlemen who were officially called to decide as to its propriety; but, although their repugnance to an attempt of this kind was not well founded, it was conscientious, and not at all blameable.

*Case* 2. This occurred recently. A man between forty and fifty years of life entered the Philadelphia Hospital in a moribund state. The abdomen was distended and contained much gas, but was not tender to the touch, nor painful to the patient; the body generally was extremely emaciated. The patient was too feeble to give any further account of himself except that he was subject to dyspepsia for some months, had frequent vomitings, and was habitually costive. Some restorative medicines were given, but the patient sank twenty-four hours after admission.

On examination after death, the whole body was found to be extremely emaciated, the abdomen much distended with gas. The contents of the thorax were in a healthy state, but on opening the abdomen the stomach was found to be extremely distended, and evidently enlarged from permanent thickening, so that the thickness of the parietes was increased, and the whole organ was at least of twice the average dimensions. It contained a pale mucus, and its internal coat was thickened and mamillated throughout the greater portion of the pyloric half, thinned by the action of the liquid near the cardiac extremity, and of a general pale slate colour. The vascular injection was extremely slight. The small intestine was also distended with mucous liquid and with gas, the parietes much thickened. Near the extremity of the ileum it dipped into a hernial sac, at the right inguinal ring. The portion of bowels contained did not exceed the size of a pigeon's egg; it was of a bluish tint, the veins upon it much distended, but not inflamed or gangrenous. The circulation being evidently retarded, but by no means interrupted. At the point of constriction where the bowels passed out of the sac, the size of the intestine was much reduced; it was not larger than that of a child of three years; the colon was also much smaller than natural, not larger than that of a patient dying of chronic dysentery; it contained some well formed fæces.

These cases are both illustrative of death following the slow symptoms of hernial structure sufficiently tight to impede the progress of fæces without destroying the circulation.

